# Clinical and Molecular Characteristics of Diabetic Retinopathy and Its Severity Complications among Diabetic Patients: A Multicenter Cross-Sectional Study

**DOI:** 10.3390/jcm11143945

**Published:** 2022-07-07

**Authors:** Hamzeh Al Zabadi, Ibrahim Taha, Rami Zagha

**Affiliations:** 1 Department of Public Health, Faculty of Medicine and Health Sciences, An-Najah National University, Nablus P.O. Box 7, Palestine; 2 Public Health Program, Faculty of Graduate Studies, An-Najah National University, Nablus P.O. Box 7, Palestine; ibrahimai.taha01@universitadipavia.it; 3 Department of Molecular Medicine, University of Pavia, 27100 Pavia, Italy; 4 Microbiology, Immunology and Pathology Department, Faculty of Medicine and Health Sciences, An-Najah National University, Nablus P.O. Box 7, Palestine; r.zagha@najah.edu

**Keywords:** diabetic retinopathy, diabetes mellitus, visual impairment, Palestine, prevalence

## Abstract

Background: Diabetic retinopathy (DR) is a complication associated with uncontrolled DM. It is a leading preventable cause of visual impairment in the world and a cause of blindness in those under 75 years old in developing countries. We aimed to explore the prevalence and associated risk factors of DR among diabetic patients in the West Bank. Materials and Methods:A quantitative multicenter cross-sectional study was conducted in all West Bank cities. Nearly, 385 patients underwent a comprehensive eye examination in addition to blood and urine tests. A previously validated questionnaire for ocular examination classification was used together with a socio-demographic and past medical history information sheet. Results: The prevalence of all DR in the West Bank was 41.8%. The prevalence of non-proliferative diabetic retinopathy (NPDR) was 50.3% (38.5% for mild NPDR, 10.6% for moderate NPDR and 1.2% for severe NPDR). The prevalence of proliferative diabetic retinopathy (PDR) was 9.9% and 39.7% for diabetic macular edema (DME) (17.4% for mild, 15.5% for moderate and 6.8% for severe DME). The prevalence of vision-threatening PDR and DME was 49.7% for both. In a univariate analysis, DR was significantly associated with body mass index; BMI (*p* = 0.035), DM duration (*p* = 0.002), Low-density lipoprotein (LDL) (*p* = 0.034), glutamic-oxaloacetic transaminase (GOT) level (*p* = 0.016) andblood urea (BU) (*p* = 0.044). A multivariate analysis showed a strong significant association between DR andpatients who had DM for 10-19years (adjusted odds ratio; AOR (95%CI); 1.843 (1.05–3.22)), abnormal levels of LDL (AOR (95%CI); 0.50 (0.30–0.83)), abnormal levels of GOT (AOR (95%CI); 0.49 (0.27–0.89)), and overweight (AOR (95%CI); 0.39 (0.19–0.80)). Conclusions: We found that the prevalence of DR in Palestine was higher than the global prevalence. Referral coordination between ophthalmologists and internal physicians is necessary to better follow up with DR patients. An interventional educational program by clinicians and public health professionals is recommended.

## 1. Introduction

Diabetes mellitus (DM) can seriously impact quality of life. There have been warnings from the World Health Organization (WHO) that the number of diabetic patients is quickly rising [1]. Studies have shown that the prevalence of DM worldwide was 9.3% (463 million) in 2019, and it is expected to rise to 10.2% (578 million) by 2030 and 10.9% (700 million) by 2045 [2]. DM is an important health problem due to its high morbidity and mortality. The presence of chronic diabetes complications increases the disease’s burden and high expenses, with expenditure estimated to be USD 760 billion in 2019 [3]. Moreover, it is among the top ten causes of death in adults, with an estimated four million deaths worldwide in 2017 [2]. In Palestine, DM is the fourth leading cause of mortality, with a prevalence of 9.1% among patients aged 20–79 years [4]. The three main types of diabetes are type 1 and type 2 DM, and gestational diabetes mellitus. Hypercalcemia and other risk factors contribute to the development of macrovascular involving the cardiovascular and peripheral vascular system, and microvascular involving neuropathy, nephropathy and retinopathy [5].

Diabetic retinopathy (DR) is a common microvascular complication. It is considered one of the significant complications associated with poor DM control and it is a leading cause of preventable visual impairment worldwide. However, treatment and early detection could help overcome additional ocular complications [6].

The global prevalence of DR in 2021 is estimated to be 10.5% (536.6 million people) and is expected to increase to 12.2% (783.2 million) in 2045 [7]. It is the primary cause of vision loss in people under the age of 75 in developed countries [8]. DR is a progressive disease affecting the integrity of microscopic vessels in the retina [9]. The stages of DR are varied and depend on the severity. However, the stages of DR according to the Early Treatment Diabetic Retinopathy Study are non-proliferative diabetic retinopathy (NPDR), proliferative diabetic retinopathy (PDR) and diabetic macular edema (DME) [8]. At the early subclinical stages of NPDR, hyperglycemia leads to the development of neurodegeneration, vascular endothelial defects and intraretinal dot hemorrhages. At this stage, DR may cause no symptoms or only very mild vision problems. PDR, a more advanced stage of DR, is characterized by severe retinal ischemia due to hypoxia, and may lead to neovascularization. At this stage, affected patients may experience severe visionloss due to significant vitreous hemorrhages, alteration in retinal permeability or retinal detachment in severe cases [10]. DME is the most common cause of visual impairment, and is characterized by macular involvements including thickening or swelling of the sub- and intra-retinal accumulation of fluids triggered by the breakdown of the blood-retinal barriers. DME can occur at any stage of DR, causing image distortion and a loss of visual acuity [11]. Therefore, DR is a serious public health issue and its early diagnosis with effective management is crucial. Current DR therapy options include intravitreal pharmacologic medications, laser photocoagulation and vitreous surgery, all therapies aiming to manage microvascular problems. Anti-VEGF agents administered intravitreally are currently the mainstay of treatment for both early and severe stages of DR [12].

Studies have shown that the development of DR is robustly correlated with hyperglycemia, higher hemoglobin A1c (HbA1c) levels, high blood pressure and longer diabetes duration [13,14]. Other risk factors that have been reported include high body mass index (BMI), dyslipidemia, nephropathy, type of DM and smoking [15,16,17,18].

The present study aimed to assess the prevalence of DR among diabetic patients in the West Bank, Palestine, and to evaluate its associated socio-demographic, clinical and biochemical characteristics.

## 2. Materials and Methods

### 2.1. Study Design, Population, Settings and Sample Size

The study design, population and sample calculation have been described elsewhere [19]. Briefly, a multicenter cross-sectional study was performedin the period between January to December 2019. According to the Palestinian Ministry of Health (PMoH), the total number of patients with DM in the West Bank, Palestine, in 2019 was estimated to be around 278,302 (MoH, 2020). All adults and elderly patients (18 years of age and above) who regularly visited and were treated in the PMoH primary health care centers in all West Bank governorates were included. Patients who refused to participate or who hadany cognitive disabilities were excluded. According to the Palestinian Central Bureau of Statistics (PCBS, 2019), 422 out of 608 primary health care centers (PHC) in the West Bank are provided by PMoH, making it the major healthcare provider for DM patients. The majority of diabetic patients visit these health care centers because they are usually insured by the Palestinian national insurance.

To calculate the sample size, we used the Epi Info™ statistical program, version 7 (CDC, Atlanta, GA, USA; https://www.cdc.gov/epiinfo/index.html (accessed on 7 January 2019)). A 95% confidence level with a type I error of 5% was assumed. In addition, we used the expected proportion (*p* = 0.5) in the population in order to have the highest sample size. Using the previous values, the total sample was calculated to be 385.

### 2.2. Data Collection

The procedure for thedata collection has been previously described [19,20]. Briefly, a list of diabetic patients was obtained from MoH in each governorate. A random sampling technique was used in which all subjects were randomly selected from the main eleven PHC centers in all West Bank governorates including Jenin, Tulkarm, Qalqelia, Nablus, Salfit, Tubas, Ramallah, Bethlehem, Jericho, Hebron and Jerusalem. Then, the selected patients were contacted by phone and informed of their participation in the study. The subjects were informed in each directorate regarding the time of their visit to the PHC center. At their attendance, and before participation, written-signed informed consent was obtained from each patient.

The selected patients were first interviewed for ten minutes to fill a previously used questionnaire that included questions about socio-demographic information and medical history [21,22]. The ocular examination part was a validated guideline according to the Early Treatment Diabetic Retinopathy Study (ETDRS) [23,24]. It should also be noted that the socio-demographic, ocular history, and biochemical parts of the questionnaire used in this study were used in previous studies [21,22] with minor modifications to adapt it to the Palestinian context.

After that, blood and urine samples were collected by a laboratory specialist. Then, all the patients underwent pupil dilation using a mydriatic agent as previously described [19,20]. A comprehensive ocular exam was performed after 20 to 30 min by an ophthalmologist using *Top-Con Slit-Lamp* and 90 diopters (D) volk optical lenses. The ocular findings were reported based on the ETDRS classification of diabetic retinopathy forpresentations of NPDR, PDR and DME [23,24].

The laboratory specialists performed bloodand urine laboratory tests. The blood (serum) tests included: glycated hemoglobin (HbA1c), alkaline phosphatase (ALK PHOS), glutamic-oxaloacetic transaminase (GOT), glutamic-pyruvic transaminase (GPT), triglycerides (TG), high-density lipoprotein (HDL), low-density lipoprotein (LDL), cholesterol (CHOL) and creatinine (CREAT). The urine tests included: microalbuminuria (MALB) and blood urea (BU).

### 2.3. Laboratory Tests Procedure

Urine and blood samples were collected from the subjects as per the company guidelines and recommendations. The full laboratory procedure of the tests has been described elsewhere [19,20]. Briefly, the subjects were asked for fast 8–10 h and then were sampled for blood using vacutainer tubes of 4 mL clot activator and 3 mL EDTA. About 50 mL of urine sample was taken as random midstream. We analyzed the urine and serum through the *HumaStar 200 system* (*Human Germany*).

It should also be noted that the methods of laboratory test analysis have been prescribed elsewhere in detail [19,20]. These methods were conducted for CHOL, TG, HDL, LDL, ALK PHOS, GOT, GPT, BU, CREAT and MALB [19,20].

The kits were manufactured by *Human Germany* and were used accordingly. The cutoff values were determined from the kit sheets for every single test together with internal and external controls. All these values and protocols have been described in detail elsewhere [19,20].

### 2.4. Ethical Consideration

Before collecting any data, the Institutional Review Board (IRB) of An-Najah National University approved the experimental procedure including the human data and samples of the study, with an ethical code number of (14 October 2016) and an approval date of the 14 December 2016. The study was also approved by the scientific research board council of the graduate studies faculty at An-Najah National University, Nablus-Palestine. In addition, the permission of work was obtained from the Palestinian MoH. All the patients signed a written consent form before their participation.

### 2.5. Statistical Analysis

IBM Statistical Product and Service Solutions (SPSS V.25) was used for all statistical analyses. We used descriptive statistical analysis to measure the mean ± standard deviation (SD) for all quantitative variables and the percentage of categorical data. Chi-square (χ^2^) and Fisher exact tests were used to determine the association between the categorical independent variables and the dependent variables. Multivariate logistic regression was performed to evaluate the association between the dependent variables and the associated independent variableswith AOR and a 95%CI using the enter method. The associations were considered statistically significant when *p*-value < 0.05.

## 3. Results

### 3.1. Socio-Demographic and Medical History Characteristics of Study Participants

The mean of age was 56.48 ± 12.337 years, with 36.4% in the age category of 55–64 years. Of all, 52.7% were females. About 56.1% of all subjects were from northern regions of the West Bank. More than half of the patients (51.9%) had primary education.

The mean BMI was 30.3 ± 5.6. About 38.4% were overweight and 49.6% were obese. The mean of DM duration was 9.6 ± 7.8 years. The vast majority of the patients had type 2 DM with 92.5%, whereas 7.5% only had type 1 DM.

Almost 46.2% were treated with oral hypoglycemic agents and 25.5% with insulin. More than half (55.3%) had hypertension and 12.7% had a history of systemic steroid therapy. In this study, approximately 20.5% were current smokers and about 10.6% had previous ocular trauma.

### 3.2. Biochemical Characteristics of Study Participants

The mean of HbA1c for all diabetic patients was 8.2 ± 1.8, whereas the means for normal DM patients and DR patients were 7.9 ± 2.0 and 8.8 ± 1.7, respectively. About 68.1% had non-controlled HbA1c levels. The GT mean was 189.6 ± 132.3 mg/dL, and nearly 50.1% had abnormal TG levels. The HDL mean was 48.6 ± 14.5 mg/dL, with 83.9% having abnormal levels. The results showed that the ALK PHOS mean was 229.8 ± 114.8 U, with 93.8% having abnormal levels. Furthermore, the CREAT mean was 1.02 ± 0.47 mg/dL and about 41.0% had abnormal levels. The results showed that the mean of BU was 34.2 ± 18.9 mg/dL and only 12.7% had abnormal levels. Lastly, the MLAB mean was 68.5 ± 78.1 mg/dL; slightly more than half (54.8%) had microalbuminuria and only 2.1% had macroalbuminuria.

### 3.3. Prevalence of Diabetic Retinopathy

The prevalence of DR among diabetic patients was found to be 41.8%. The prevalence of NPDR was 50.3% (38.5% mild, 10.6% moderate and 1.2% severe). The prevalence of PDR was 9.9%, while 39.7% was the prevalence of DME (17.4% mild, 15.5% moderate and 6.8% severe). The prevalence of vision-threatening PDR and DME was 49.7% for both.

### 3.4. Diabetic Retinopathy Patients’ Characteristics

Of the DR patients, 42.6% were from the northern governorates. About 51% of all DR patients were females and almost 40% (*n* = 64) were in the 55–64 age group. It was found that 54% of the DR patients had primary education. The results showed a statistically significant association between DR and BMI (*p* = 0.03). About 149 of 161 DR patients had type 2 DM (41.9%). A statistically significant association between DR and DM duration (*p* = 0.002) was found. Table 1 shows the univariate analysis of the socio-demographic, medical, and ocular characteristics of diabetic patients.

The study results revealed statistically significant associations between DR and LDL (*p* = 0.034), GOT level (*p* = 0.016) and BU (*p* = 0.044). No statistically significant association between DR and HDL was found. Regarding albuminuria, the majority of patients (53.4%) had microalbuminuria and only three (1.9%) had macroalbuminuria. Table 2 shows the univariate analysis of the laboratory characteristics of diabetic patients.

### 3.5. NPDR and Vision-Threatening Patient Characteristics

The results showed that 51.6% of patients withthreatened vision were females. Obesity was found in 34 patients with threatened vision and 50.3% of these patients had type 2 DM. About 58% of all patients had DM for more than 10 years. Chi-square testing illustrated a statistically significant association between NPDR and threaten vision with directorates (*p* = 0.028) and systemic steroid therapy (*p* = 0.02). No statistically significant associations between NPDR, threatened vision and the laboratory findings were found. However, it should be noted that only eight (0.04%) patients with NPDR and threatened vision had normal levels of alkaline phosphatase. Table 3 illustrates the univariate analysis for NPDR and threatened vision (PDR, DME).

### 3.6. Regression Analysis for Factors Associated with Diabetic Retinopathy

A multivariate binary logistic regression showed that DR was associated with 10–19 years DM duration (AOR, 95%CI; 1.84, 1.05–3.22), abnormal levels of LDL (AOR, 95%CI; 0.50, 0.30–0.83), abnormal levels of GOT (AOR, 95%CI; 0.49, 0.27–0.89) and overweight (AOR, 95%CI; 0.39, 0.19–0.80). Table 4 shows the multivariate logistic regression for factors associated with diabetic retinopathy.

### 3.7. Regression Analysis for Factors Associated with NPDR and Threatened Vision

A multivariate binary logistic regression showed that patients who had systemic steroid therapy (AOR, 95%CI; 2.94, 1.05–8.02) and abnormal levels of CHOL (AOR, 95%CI; 2.94, 1.05–8.02) had a higher risk ofdevelopingthreatened vision. Table 5 illustrates multivariate logistic regression for factors associated with NPDR and threatened vision (PDR, DME).

## 4. Discussion

The main finding of our study was that the prevalence of any DR among Palestinians with DM was 41.8%. This is higher than the global prevalence (34.6%) [25]. We reported a higher prevalence of DR compared to North America, the UK, Italy, China and Germany (33.3%, 36.6% 27.6%, 27.9%and 25.8%, respectively) [26,27,28,29,30]. On the other hand, we reported a lower DR prevalence compared to France and Norway (50.1% and 61%, respectively) [31,32].

The variations in DR prevalence among different populations could be explained by the differences in the characteristics of each population, as well as the methods and criteria used for measuring the DR prevalence. ForPalestine, we believe that the most significant contributing factor could be poor glycemic control [33]. Although not significant, we noticed that the majority (*n* = 88) of DR patients (*n* = 161) had only a primary education. Given the importance of self-management and knowledge about the mechanism behind DM and DR, a possible explanation is that this group of participants might have poor knowledge about the mechanism of occurrence of their disease and thus experiencepoor control of their conditions due to their low level of education.

In the present study, we found a significant association between all DR and BMI, GOT, LDL, duration of DM, and BU. This is in accordance with the global prevalence and the major risk factors of diabetic retinopathy study [15]. Unlike our findings, different studies reported a significant association between DR and HbA1c [15,34]. Previous studies highlighted that being overweight and obese were risk factors for DM [35]. BMI and its association with DR were described in different studies. For example, Rita Laiginhas et al. reported that high BMI had a significant association with PDR but not all DR [36]. Contrary to our findings, Yue Zhou et al. showed that neither being obese nor being overweight was correlated with anincreased risk of DR. It should be noted, however, that a significant increase in HDL and blood pressure and a significant decrease in HbA1c were observed in individuals with higher BMIs (all of which are risk factors for DR) [37]. A few published studies explained the relationship between DR and LDL levels. A study conducted by Ronald Klein and colleagues did not provide evidence for any association between increasing levels of LDL and the incidence of DR [38].

Another study was conducted in South Africa by Farzana Gan et al. which reported a strong association between the presence of DR and high levels of LDL [39].Blood urea nitrogen (BU) values are usually used to evaluate kidney function. In our study, we reported that BU levels were significantly associated with DR. Our findingsaresimilar to those from studies conducted in Sudan and China [29,40]. No previous studies considered a GOT as a risk for DR. Our study was the first to describe the relation between GOT and all DR. In the meantime, we recommend further studies to clarify such a relationship.

The multivariate analysis revealed that patients who had systemic steroid therapy and abnormal levels of CHOL were at increased risk ofdevelopingthreatened vision (PDR and DME). Contrary to our results, other studies have shown the efficacy of systemic steroids in preventing DR onset and reversing early retinopathy and/or slowing the progression of retinopathy [41]. A meta-analysis of sevenstudies did not find obvious variations in levels of CHOL between DR patients and controls. This is in accordance with our findings, as slightly higher LDL levels were observed in the DR cases [37]. One of the possible explanations for these differences across the globe could be related to genetic variations. Further studies are needed to investigatethis further.

In Palestine, limited studies have beenconducted. A retrospective medical records-based study based on patient records was conducted in Gaza by Ayman M. AbuMustafa aimed atevaluating the clinical/biochemical associations with diabetic retinopathy. In this study, only males with type 2 diabetes aged 40–60 years old were included [21]. However, our study was more comprehensive by including the two DM types in both genders, the sample size used in our study was higher, and the geographic distribution of our study was wider by including all West Bank regions and Jerusalem.

Our study was the first nationwide study that evaluated DR and its clinical and biochemical characteristics together with its severity levels among diabetic patients. Moreover, our study population represented the majority of DM patients in the West Bank, as the PHC centers of MoH are the main healthcare providers (MoH, 2017). In the meantime, comprehensive eye examinations of all patients were performed by the same ophthalmologist. Importantly enough, this study did not use previous medical records to extract laboratory tests for patients, but all laboratory tests reported in this study were performed using one standard analyzer in the same laboratory. Every epidemiological study could have some limitations. Our study might be limited in that the ocular and medical information wastaken from the patients only through a face-to-face interview without depending on medical records. The absence of a baseline history among the subjects before they werediagnosed with DM might have affected our results regarding the relation between retinopathy formation and DM.

## 5. Conclusions

We found that the prevalence of DR in Palestine was higher than the global prevalence.As DR is considered one of the major complications of DM, ophthalmologists and other eye care providers should give more practical attention to some biochemical findings in following the patients with DRwho usually seek ocular evaluation in their clinics rather than depending on ocular examination alone. For this, referral coordination between ophthalmologists and internal physicians is necessary to better follow up with those patients. An interventional educational program by clinicians and public health professionals is recommended.

## Figures and Tables

**Table 1:** Univariate analysis of the socio-demographic, medical, and ocular characteristics of diabetic patients.

Variables	Absence of DR	Presence of DR	*p*-Value *
	N (%)	N (%)	
**Directorate categories**			0.554
North of West Bank	124 (57.4)	92 (42.6)	
Middle of West Bank	37 (54.4)	31 (45.6)	
South of West Bank	63 (62.4)	38 (37.6)	
**Gender**			0.696
Female	120 (59.1)	83 (40.9)	
Male	104 (57.1)	78 (42.9)	
**Age categories**			0.602
≤44 Years	26 (57.8)	19 (42.2)	
45–54 Years	63 (63.0)	37 (37.0)	
55–64 Years	76 (54.3)	64 (45.7)	
≥65 Years	59 (59.0)	41 (41.0)	
**Education level**			0.473
Not educated	21 (56.8)	16 (43.2)	
Primary education	112 (56.0)	88 (44.0)	
Secondary education	45 (57.0)	34 (43.0)	
High education	46 (66.7)	23 (33.3)	
**BMI categories**			0.035
Underweight	0 (0.0)	2 (100.0)	
Normal weight	19 (43.2)	25 (56.8)	
Overweight	94 (63.5)	54 (36.5)	
Obesity	111 (58.1)	80 (41.9)	
**DM types**			0.96
Type 1 DM	17 (58.6)	12 (41.4)	
Type 2 DM	207 (58.1)	149 (41.9)	
**DM duration categories**			0.002
≤4 Years	85 (67.5)	41 (32.5)	
5–9 Years	52 (66.7)	26 (33.3)	
10–19 Years	65 (49.2)	67 (50.8)	
≥20 Years	22 (44.9)	27 (55.1)	
**Hypertension**			0.523
Absent	97 (56.4)	75 (43.6)	
Present	127 (59.6)	86 (40.4)	
**Current smoking**			0.805
No	179 (58.5)	127 (41.5)	
Yes	45 (57.0)	34 (43.0)	
**Systemic steroid therapy**			0.64
No	197 (58.6)	139 (41.4)	
Yes	27 (55.1)	22 (44.9)	
**Ocular trauma**			0.292
No	197 (57.3)	147 (42.7)	
Yes	27 (65.9)	14 (34.1)	
**Topical steroid therapy**			0.299
No	213 (58.8)	149 (41.2)	
Yes	11 (47.8)	12 (52.2)	
**Retinopathy treatment**			0.073
No	197 (60.1)	131 (39.9)	
Yes	27 (47.4)	30 (52.6)	

DR: diabetic retinopathy; BMI: body mass index; DM: diabetes mellitus, * χ^2^-test.

**Table 2:** Laboratory characteristics of diabetic patients.

Variables	Absence of DR N (%)	Presence of DR N (%)
**HbA1c ^(1)^**		
Controlled	80 (65.0)	43 (35.0)
Non-controlled	144 (55.0)	118 (45.0)
**TG ^(2)^**		
Normal	111 (57.8)	81 (42.2)
Abnormal	113 (58.5)	80 (41.5)
**CHOL ^(3)^**		
Normal	136 (57.6)	100 (42.4)
Abnormal	88 (59.1)	61 (40.9)
**HDL ^(4)^**		
Normal	39 (62.9)	23 (37.1)
Abnormal	185 (57.3)	138 (42.7)
**LDL ^(5)^**		
Normal	155 (55.0)	127 (45.0)
Abnormal	69 (67.0)	34 (33.0)
**ALKPHOS ^(6)^**		
Normal	16 (66.7)	8 (33.3)
Abnormal	208 (57.6)	153 (42.4)
**GOT ^(7)^**		
Normal	173 (55.3)	140 (44.7)
Abnormal	51 (70.8)	21 (29.2)
**GPT ^(8)^**		
Normal	206 (57.5)	152 (42.5)
Abnormal	18 (66.7)	9 (33.3)
**CREAT ^(9)^**		
Normal	137 (60.4)	90 (39.6)
Abnormal	87 (55.1)	71 (44.9)
**BU ^(10)^**		
Normal	202 (60.1)	134 (39.9)
Abnormal	22 (44.9)	27 (55.1)
**Albuminuria**		
Normal microalbuminuria	94 (56.6)	72 (43.4)
Microalbuminuria	125 (59.2)	86 (40.8)
Macroalbuminuria	5 (62.5)	3 (37.5)

^(1)^: Glycated hemoglobin; ^(2)^: triglycerides; ^(3)^: cholesterol; ^(4)^: high-density lipoprotein; ^(5)^: low-density lipoprotein; ^(6)^: alkaline phosphatase; ^(7)^: glutamic-oxaloacetic transaminase; ^(8)^: glutamic-pyruvic transaminase; ^(9)^: creatinine; ^(10)^: blood urea. The normal cutoff values for each test were obtained from kit sheets as the following: CHOL ≤ 190 mg/dL; TG ≤ 150 mg/dL; HDL ≥ 60 mg/dL; LDL ≤ 129 mg/dL; ALK PHOS ≤ 104 U/L, 129 U/L for females and males, respectively; GOT ≤ 31 U/L, 35 U/L for females and males, respectively; GPT ≤ 34 U/L, 45 U/L for females and males, respectively; BU ≤ 50 mg/dL; CREAT ≤ 0.9 mg/dL, 1.1 mg/dL for females and males, respectively; MLAB 0–30 mg/L; HbA1c ≤ 7% (NGSP5/DCCT6) for glycemic control.

**Table 3:** Univariate analysis for Non-proliferative diabetic retinopathy (NPDR) and threatened vision (Proliferative diabetic retinopathy; PDR, diabetic macular edema; DME).

Variables	NPDR N (%)	Threatened Vision (PDR, DME) N (%)	*p*-Value *
**Directorates categories**			0.028
North of West Bank	43 (46.7)	49 (53.3)	
Middle of West Bank	12 (38.7)	19 (61.3)	
South of West Bank	26 (68.4)	12 (31.6)	
**Gender**			0.811
Female	41 (49.4)	42 (51.6)	
Male	40 (51.3)	38 (48.7)	
**Age categories**			0.351
≤44 Years	13 (68.4)	6 (31.6)	
45–54 Years	16 (43.2)	21 (56.6)	
55–64 Years	32 (50.0)	32 (50.0)	
≥65 Years	20 (48.8)	21 (51.2)	
**Education level**			0.406
Not educated	11 (64.7)	6 (35.3)	
Primary education	39 (44.8)	48 (55.2)	
Secondary education	19 (55.9)	15 (44.1)	
High education	12 (52.2)	11 (47.8)	
**BMI categories** **^(1)^**			0.672
Underweight	1 (50.0)	1 (50.0)	
Normal weight	13 (52.0)	12 (48.0)	
Overweight	31 (56.4)	24 (43.6)	
Obesity	36 (45.6)	43 (54.4)	
**DM types**			0.563
Type 1 DM	7 (58.3)	5 (41.7)	
Type 2 DM	74 (49.7)	75 (50.3)	
**DM duration categories**			0.747
≤4 Years	21 (51.2)	20 (48.8)	
5–9 Years	16 (59.3)	11 (40.7)	
10–19 Years	31 (47.0)	35 (53.0)	
≥20 Years	13 (48.1)	14 (51.9)	
**Hypertension**			0.481
Absent	35 (47.3)	39 (52.7)	
Present	46 (52.9)	41 (47.1)	
**Current smoking**	60 (47.2)		0.133
No	21 (61.8)	67 (52.8)	
Yes		13 (38.2)	
**Systemic steroid therapy**			0.02
No	75 (54.0)	64 (46.0)	
Yes	6 (27.3)	16 (72.7)	
**Ocular trauma**			0.274
No	72 (49.0)	75 (51.0)	
Yes	9 (64.3)	5 (35.7)	
**Topical steroid therapy**			0.928
No	75 (50.3)	74 (49.7)	
Yes	6 (50.0)	6 (50.0)	
**HbA1c** **^(2)^**			0.876
Controlled	22 (50)	22 (50)	
Non-controlled	59 (50.4)	58 (49.6)	
**TG** **^(3)^**			0.432
Normal	38 (46.9)	43 (53.1)	
Abnormal	43 (53.8)	37 (46.3)	
**CHOL** **^(4)^**			0.051
Normal	22 (50)	22 (50)	
Abnormal	59 (50.4)	58 (49.6)	
**HDL** **^(5)^**	11 (47.8)		0.825
Normal	70 (50.7)	12 (52.2)	
Abnormal		68 (49.3)	
**LDL** **^(6)^**			0.176
Normal	68 (53.1)	60 (46.9)	
Abnormal	13 (39.4)	20 (60.6)	
**ALK PHOS** **^(7)^**			0.167
Normal	2 (25)	6 (75)	
Abnormal	79 (51.6)	74 (48.4)	
**GOT** **^(8)^**			0.474
Normal	69 (48.9)	72 (51.1)	
Abnormal	12 (60)	8(40)	
**GPT** **^(9)^**			0.167
Normal	74 (48.7)	78 (51.3)	
Abnormal	7 (77.8)	8 (22.2)	
**CREAT** **^(10)^**			0.342
Normal	42 (46.7)	48 (53.3)	
Abnormal	39 (54.9)	32 (54.1)	
**BU** **^(11)^**			0.972
Normal	68 (50.4)	67 (49.6)	
Abnormal	13 (50)	13 (50)	
**Albuminuria**			0.742
Normal microalbuminuria	38 (52.8)	34 (47.2)	
Microalbuminuria	42 (48.8)	44 (51.2)	
Macroalbuminuria	1 (33.3)	2 (66.7)	

^(1)^:Body mass index; ^(2)^: glycated hemoglobin; ^(3)^: triglycerides; ^(4)^: cholesterol; ^(5)^: high-density lipoprotein; ^(6)^: low-density lipoprotein; ^(7)^: alkaline phosphatase; ^(8)^: glutamic-oxaloacetic transaminase; ^(9)^: glutamic-pyruvic transaminase; ^(10)^: creatinine; ^(11)^: blood urea. * χ^2^-test.

**Table 4:** Multivariate logistic regression for factors associated with diabetic retinopathy.

Variables	aOR ^(1)^	95%CI ^(2)^	*p*-Value
**DM duration**			
5–9 Years	1.034	0.55–1.931	0.917
10–19 Years	1.843	1.054–3.220	0.032
≥20 Years	2.005	0.913–4.403	0.083
≤4 Years ^#^			
**HbA1c** **^(3)^**	1.416	0.866–2.316	0.166
Non-controlled			
Controlled ^#^			
**LDL** **^(4)^**			0.008
Abnormal	0.501	0.301–0.835	
Normal ^#^			
**GOT** **^(5)^**			0.019
Abnormal	0.494	0.274–0.890	
Normal ^#^			
**BU** **^(6)^**			0.103
Abnormal	1.709	0.897–3.254	
Normal ^#^			
**BMI** **^(7)^**			
Overweight	0.393	0.193–0.801	0.01
Obesity	0.572	0.285–1.148	0.116
Normal weight ^#^	–	–	

^(1)^: Adjusted odds ratio; ^(2)^: (95)% confidence intervals; ^(3)^: glycated hemoglobin; ^(4)^: low-density lipoprotein; ^(5)^: glutamic-oxaloacetic transaminase; ^(6)^: blood urea; ^(7)^: body mass index; ^#^: reference category (aOR = 1).

**Table 5:** Multivariate logistic regression for factors associated with NPDR and threatened vision (PDR, DME).

Variables	aOR ^(1)^	95%CI ^(2)^	*p*-Value
**Directorates categories**			
Middle of West Bank	1.255	0.531–2.965	0.605
South of West Bank	0.459	0.202–1.044	0.063
North of West Bank ^#^			
**Systemic steroid therapy**			
Yes	2.948	1.059–8.209	0.039
No ^#^			
**CHOL** **^(3)^**	2.002	1.021–3.926	0.043
Normal			
Abnormal^#^			

^(1)^: Adjusted odds ratio; ^(2)^: 95% confidence intervals; ^(3)^: cholesterol levels; ^#^:reference category (aOR = 1).

## Data Availability

The datasets generated and/or analyzed during the current study are not publicly available due to privacy and confidentiality but are available from the corresponding author upon reasonable request.

## Abbreviations

BMI	Body mass index
DM	Diabetes mellitus
DR	Diabetic retinopathy
ETDRS	Early treatment diabetic retinopathy study
NPDR	Non-proliferative diabetic retinopathy
PDR	Proliferative diabetic retinopathy
SPSS	Statistical package for social sciences
VTDR	Vision-threatening diabetic retinopathy
WHO	World Health Organization
HbA1c	Glycated hemoglobin
TG	Triglycerides
CHOL	Cholesterol
HDL	High-density lipoprotein
LDL	Low-density lipoprotein
ALK PHOS	Alkaline phosphatase
GOT	Glutamic-oxaloacetic transaminase
GPT	Glutamic-pyruvic transaminase
CREAT	Creatinine
BU	Blood urea
MoH	Ministry of Health
PHC	Primary health centers
OR	Odds ratio
CI	Confidence intervals
